# Association between rotator cuff muscle cross-sectional area and dorsal scapular artery blood flow velocity in patients with rotator cuff tears

**DOI:** 10.1016/j.xrrt.2025.08.014

**Published:** 2025-09-08

**Authors:** Keita Kawabuchi, Masatoshi Nakamura

**Affiliations:** a Institute for Rehabilitation Room, Tottori Prefectural Central Hospital, Tottori, Japan; b Faculty of Rehabilitation Science, Nishi Kyushu University, Kanzaki, Saga, Japan

**Keywords:** Rotator cuff tears, Muscle cross-sectional area, Peak systolic velocity, Dorsal scapular artery, Magnetic resonance imaging, Ultrasound imaging

## Abstract

**Background:**

Atrophy of the rotator cuff (RC) muscles in the context of in patient's RC tears often results in humeral instability and scapular compensation, leading to symptoms like pain. Moreover, despite reports of increased in the peak systolic velocity (PSV) of the dorsal scapular artery (DSA) as a contributing factor, the specific relationship between RC atrophy and these hemodynamic changes has not been elucidated. This study aimed to quantitatively investigate the association between the cross-sectional area (CSA) of RC muscles and the PSV of the DSA.

**Methods:**

Thirty-eight patients with RC tears were included. The CSA of the supraspinatus, infraspinatus (ISP), and subscapularis muscles were measured on T1-weighted magnetic resonance images at the glenoid face (GF) and scapular spine levels. The PSV of the DSA was assessed by Doppler ultrasonography. Statistical analyses were conducted using linear mixed models (LMM) and multiple regression analysis, adjusting for age and body mass index as covariates.

**Results:**

A significant negative interaction between ISP-CSA and PSV was observed at the GF level in the LMM (*P* = .03). Multiple regression analysis further indicated that only the ISP-CSA at the GF level exhibited a significant negative association with PSV (*R*^*2*^ = 0.23, *P* = .03). No significant associations were observed for other muscles. At the scapular spine level, neither LMM nor multiple regression analysis identified any significant interactions or effects.

**Conclusion:**

These findings suggest that a decreased CSA of the ISP at the GF level is associated with increased PSV of the DSA, indicating that RC muscle atrophy may influence local hemodynamics in the periscapular region.

Rotator cuff tears (RCTs) are among the most common pain disorders of the shoulder, with various reported risk factors, such as high blood glucose, hypertension, body weight, and smoking habits.^33^ Among the factors that influence the clinical outcomes in RCTs, the volume of the rotator cuff (RC) muscles plays a critical role. Muscle atrophy occurring in the early stages of RCTs is thought to result from decreased voluntary muscle contraction due to pain and mechanical unloading, further exacerbated by the activation of catabolic pathways via inflammatory signals.^9^ RC muscle atrophy and degeneration have been associated not only with poorer surgical outcomes following arthroscopic RC repair but also with reduced success rates in conservative management.^10^^,^^29^

Moreover, the condition of the RC muscles is thought to affect scapular kinematics. A 3-dimensional motion analysis study evaluating the relationship among pain, tear size, and scapulohumeral rhythm in patients with RCT reported that larger tears were associated with restricted glenohumeral motion and relatively increased scapular upward rotation, leading to high scapulohumeral rhythm values.^27^ This finding implies that larger tears may lead to reduced structural support in the glenohumeral joint, necessitating greater reliance on scapular motion. Moreover, compared with patients with partial tears or subacromial pain syndrome, those with large RCTs exhibited significantly greater scapular upward rotation during forward elevation and abduction.^19^ These findings suggest that RC atrophy may contribute to scapular dyskinesis.

Interestingly, rehabilitation that focused on scapular intervention was reported to alleviate pain and improve shoulder function in patients with RCTs through enhanced muscle flexibility, strengthening, and proprioception.^30^ However, conservative treatment rarely leads to significant improvement in scapular positioning abnormalities, and the mechanisms through which scapular-focused interventions positively influence pain and function in RCTs remain unclear.^3^ A recent study begun to explore hemodynamic factors as potential explanatory mechanisms. In painful RCTs, increased peak systolic velocity (PSV) of the anterior humeral circumflex artery is associated with high levels of inflammatory cytokines and nocturnal pain.^2^^,^^31^ Furthermore, increased PSV of the dorsal scapular artery (DSA), which supplies the periscapular tissues, was reported in painful RCTs and significantly associated with shoulder active range of motion.^18^ Hemodynamic alterations have been reported in several orthopedic conditions. For instance, increased PSV has been observed in the genicular arteries of patients with knee osteoarthritis^8^ and in the lumbar arteries of individuals with low back pain.^7^ These observations indicate a general tendency for PSV to be elevated on the symptomatic side. The increased PSV observed in the DSA of shoulders with RCTs is therefore likely to reflect underlying pathophysiologic changes, although the precise mechanism remains unclear. Interestingly, an anatomical variation has been reported in which the DSA also supplies blood flow to the RC muscles.^13^^,^^23^^,^^32^ Therefore, an increase in the PSV of the DSA may indirectly alter the blood supply to these muscles, potentially promoting RC muscles atrophy and thus reflecting their physiological status.

No study has quantified the relationship between RC muscle volume and DSA-PSV. This study aimed to quantitatively investigate the association between RC muscle cross-sectional area (CSA) and PSV of the DSA and clarify the association between muscle volume and blood flow velocity of arteries supplying the scapular region. A better understanding of this association may contribute to the development of optimized rehabilitation strategies and therapeutic interventions in managing RCTs.

## Materials and methods

### Study design and ethics approval

This retrospective study was conducted using anonymized data in accordance with an opt-out approach and was approved by the institutional ethics committee (approval no. 2024-92).

### Participants

Patients diagnosed with RCTs at our institution between June 2021 and March 2025 were included. Patients were excluded if they had uncontrolled hypertension; clinically significant cardiovascular disease (eg, heart failure or valvular pathology under active treatment); pseudoparalysis due to massive tears; or marked hypertrophy of the trapezius/levator scapulae that precluded vascular imaging. Demographic and clinical information included age, height, weight, body mass index (BMI), and sex. The severity of tears was evaluated using the Cofield classification of tears: small (<1 cm), medium (1-3 cm), large (3-5 cm), and massive (>5 cm).^6^ Pain intensity at rest was assessed using a visual analog scale, and active range of motion of the shoulder was measured in 3 planes: anterior elevation, abduction (AB), and external rotation.

### Magnetic resonance imaging acquisition protocol

Magnetic resonance imaging (MRI) was performed using a 1.5 T scanner (MAGNETOM Aera; Siemens AG, Munich, Germany) with a dedicated shoulder coil (shoulder small 16 or shoulder large 16, Siemens AG). The patient was scanned in a lateral decubitus position, and the imaging field encompassed the target shoulder and associated RC musculature. The lateral decubitus position was chosen to center the scapula within the homogeneous region of the static magnetic field, thereby enhancing image quality and fat-suppression uniformity, and to minimize motion artifacts by keeping the scapula firmly in contact with the table. Coronal T1-weighted images were acquired with the following parameters: repetition time, 450 ms; echo time, 12 ms; bandwidth, 172 Hz/pixel; flip angle, 150°; pixel size, 0.5 × 0.5 mm; slice thickness, 3.0 mm; and field of view, 160 × 160 mm. The number of excitations was set to 2, and the scan time was 2 min and 3 s. All images were saved in Digital Imaging and Communications in Medicine format.

### Magnetic resonance imaging analysis protocol

**Figure 1:**
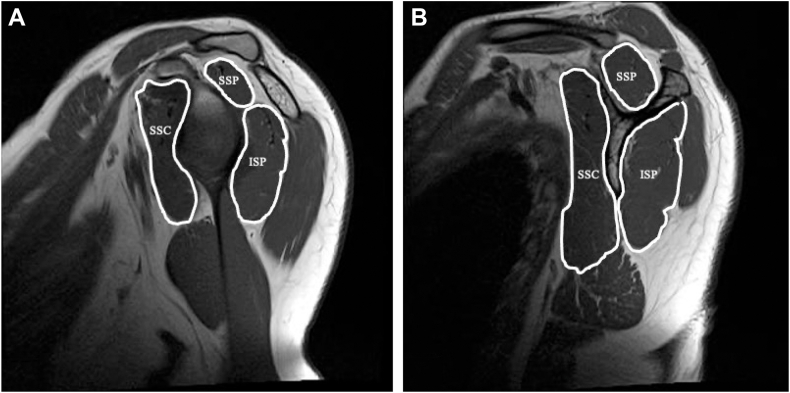
Measurement of muscle cross-sectional areas of the supraspinatus, infraspinatus, and subscapularis muscles on T1-weighted magnetic resonance images at the glenoid face and scapular spine levels. (**A**) Glenoid face level. (**B**) Scapular spine level. *ISP*, infraspinatus muscle; *SSC*, subscapularis muscle; *SSP*, supraspinatus muscle.

The CSA of the RC muscles were manually traced using the freehand region of the interest tool in Synapse Scope (Fujifilm Medical, Tokyo, Japan), carefully delineating only the muscle tissue (Fig. 1). The supraspinatus (SSP), infraspinatus (ISP), and subscapularis (SSC) were analyzed. As the ISP and teres minor could not be clearly distinguished, they were measured as a single unit. Measurements were taken at 2 anatomical levels. The glenoid face (GF) level was identified as the slice where the glenoid surface was visible.^11^ The scapular spine (SS) level was defined as the most lateral sagittal slice where the scapular spine joined the scapular body, typically corresponding to the “Y-shaped” view at the spinoglenoid notch.^21^ In this study, we evaluated the CSA at the GF level as an indicator of periglenohumeral joint stability and the CSA at the SS level as an index of overall muscle atrophy.^11^^,^^15^ A prior study demonstrated excellent interobserver reliability for MRI area measurements, with intraclass correlation coefficients (ICC) exceeding 0.96.^11^

### Assessment of the PSV of the DSA

**Figure 2:**
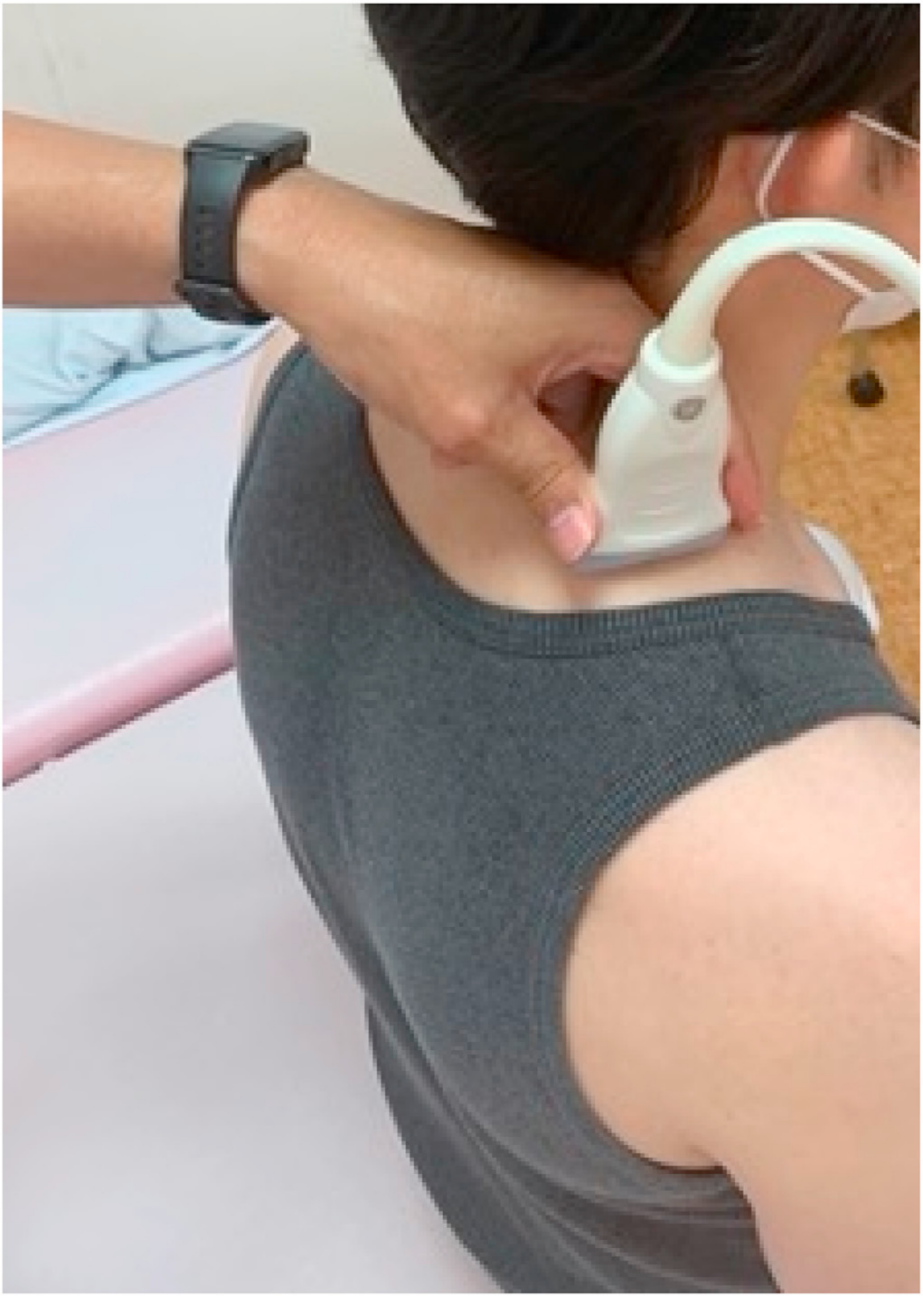
The position of the probe and position of the patient.

PSV of the DSA was assessed using a pulsed Doppler method, following the procedure described by Kawabuchi et al.^18^ An ultrasound imaging system (LOGIQ e, GE Healthcare Japan, Tokyo, Japan) was used. Participants sat comfortably with their hips and knees flexed at 90°, heads facing forward, and arms relaxed at their sides. Using a linear probe, a short-axis view of the DSA was obtained at the second rib level near the medial border of the scapula. Then, the probe was rotated to obtain a longitudinal view, and the Doppler signal was confirmed. The insonation angle was set to 60°. Once a stable waveform was obtained, the PSV was manually measured 3 times, and the average value was used for the analysis (Fig. 2). The reliability of this measurement protocol was previously validated.^18^

### Data analysis

All analyses were performed using EZR version 1.52 (Saitama Medical Center, Jichi Medical University, Saitama, Japan). A *P* value <.05 was considered significant. To assess the intrarater reliability of CSA measurements on MRI, ICC 1, 2 were calculated. For this purpose, CSA measurements of SSP, ISP, and SSC were obtained 2 times, 7 days apart, in 10 randomly selected participants at both GF and SS levels. To account for interindividual variability, exploratory analysis was performed using linear mixed models (LMM). The dependent variable was the muscle type (SSP, ISP, and SSC), and the fixed effects included the muscle type, PSV of the DSA, and interaction term (muscle type × PSV). Age and BMI were included as covariates.^12^^,^^25^ Participants were treated as a random effect to account for intersubject differences. Multiple linear regression analyses were performed to further examine the association between the RC muscle and PSV. The CSA of each muscle was treated as the dependent variable, with PSV as the independent variable, adjusted for age and BMI.

A post-hoc power analysis was conducted with G∗Power 3.1 for a multiple linear regression model that included 3 predictors and used a 2-sided α of 0.05.

## Results

In total, 42 patients were initially enrolled in this study. After excluding 2 cases in which blood flow could not be quantified and 2 cases with missing data, 38 patients were included in the final analysis. Patient characteristics are summarized in Table I and Table II.

**Table I:** Demographic and clinical information of the participants.

Variable	N = 38, Mean (SD)
Age	68.0 (7.5)
Height (cm)	162.8 (7.7)
Weight (kg)	64.4 (7.7)
BMI (kg/cm2)	24.3 (3.4)
Sex	
Male (%)	24.0 (63.0%)
Female (%)	12.0 (31.0%)
Cofield	
Small	9.0 (23.0%)
Medium	15.0 (39.0%)
Large	14.0 (36.0%)
Massive	0 (0%)
VAS (cm)	
Small	2.6 (n = 8)
Medium	2.6 (n = 11)
Large	1.4 (n = 12)
Massive	0 (n = 0)
ROM (°)	
AE	108.7 (41.1)
AB	96.3 (43.7)
ER	44.9 (20.8)

*AB*, abduction; *AE*, anterior elevation; *BMI*, body mass index; *ER*, external rotation; *ROM*, range of motion; *SD*, standard deviation; *VAS*, visual analog scale.

**Table II:** Clinical characteristics of the peak systolic velocity of the dorsal scapular artery and cross-sectional areas of related muscles.

Variable	Mean (SD)
PSV of the DSA (cm/s)	22.5 (7.3)
GF-SSP (mm^2^)	166.8 (96.5)
GF-ISP (mm^2^)	746.4 (178.2)
GF-SSC (mm^2^)	860.1 (309.7)
SS-SSP (mm^2^)	392.4 (285.4)
SS-ISP (mm^2^)	1,111.1 (257.4)
SS-SSC (mm^2^)	1,460.7 (561.1)

*SD*, standard deviation; *PSV*, peak systolic velocity; *SSP*, supraspinatus muscle; *ISP*, infraspinatus muscle; *SSC*, subscapularis muscle; *GF*, glenoid face level; *SS*, scapular spine level.

The intra-rater reliability for RC muscle CSA measurements was excellent, with the following ICC (1, 2): SSP-GF, 0.89 (95% confidence interval (CI), 0.59-0.97); ISP-GF, 0.95 (95% CI, 0.82-0.99); SSC-GF, 0.97 (95% CI, 0.90-0.99); SSP-SS, 0.94 (95% CI, 0.78-0.99); ISP-SS, 0.97 (95% CI, 0.89-0.99); and SSC-SS, 0.97 (95% CI, 0.87-0.99).

The results of the LMM analysis are shown in Table III. In the GF level, fixed-effect analysis revealed significant differences for ISP and SSC (*P* < .01), whereas SSP and PSV were not significant (SSP, *P* = .06; PSV, *P* = .86). A significant interaction was found between ISP and PSV of the DSA in the GF level (*P* = .03). In the SS level, significant differences were observed among SSP, ISP, and SSC as fixed effects (SSP, *P* = .01; ISP and SSC, *P* < .01); however, PSV showed no significant effect (*P* = .85), and no significant interaction terms were identified.

**Table III:** Results of the linear mixed model analysis.

Predictors	Glenoid face		Scapular spine	
	Estimate (SD)	*P* value	Estimate (SD)	*P* value
SSP (mm^2^)	215.9 (114.2)	.06	5,17.8 (209.4)	.01∗
ISP (mm^2^)	983.9 (114.2)	<.01∗	1,374.3 (209.4)	<.01∗
SSC (mm^2^)	1,030.6 (114.2)	<.01∗	1701.7 (209.4)	<.01∗
PSV (cm/s)	0.7 (3.7)	.86	1.3 (6.5)	.85
SSP × PSV	−2.0 (4.8)	.68	5.6 (8.8)	.53
ISP× PSV	−10.7 (4.8)	.03∗	−12.8 (8.7)	.14
SSC × PSV	−6.8 (4.8)	.15	−11.2 (8.7)	.20

*ISP*, infraspinatus muscle; *PSV*, peak systolic velocity; *SD*, standard deviation; *SSC*, subscapularis muscle; *SSP*, supraspinatus muscle.

ISP, SSC, and SSP are rotator cuff muscles; PSV denotes the peak systolic velocity measured in the dorsal scapular artery.

∗ *P* value <.05.

The results of the multiple linear regression analyses are presented in Tables IV and V. In the GF level, ISP-CSA showed a significant negative association with PSV (*R*^*2*^ = 0.23, *P* = .03). Conversely, no variables demonstrated a significant effect on PSV in the SS level.

**Table IV:** Results of multiple regression analysis for the glenoid face level.

Variable	Estimate (SD)	95% CI (lower to upper)	*P* value	Unadjusted R^2^	Adjusted R^2^
SSP (mm^2^)	−1.2 (2.1)	−5.4 to 3.1	.59	0.10	0.02
ISP (mm^2^)	−8.8 (3.7)	−16.4 to −1.2	.03∗	0.29	0.23
SSC (mm^2^)	−6.3 (7.3)	−21.3 to 8.8	.40	0.03	−0.05

*CI*, confidence interval; *ISP*, infraspinatus muscle; *SD*, standard deviation; *SSC*, subscapularis muscle; *SSP*, supraspinatus muscle.

ISP, SSC, and SSP are rotator cuff muscles.

∗ *P* value <.05.

**Table V:** Results of multiple regression analysis for the scapular spine level.

Variable	Estimate (SD)	95% CI (lower to upper)	*P* value	Unadjusted R^2^	Adjusted R^2^
SSP (mm^2^)	−4.1 (5.8)	−15.9 to 7.7	.49	0.08	−0.001
ISP (mm^2^)	−10.6 (5.5)	−21.9 to −0.6	.06	0.20	0.13
SSC (mm^2^)	−8.6 (12.7)	−34.4 to 17.2	.50	0.05	−0.03

*CI*, confidence interval; *ISP*, infraspinatus muscle; *SD*, standard deviation; *SSC*, subscapularis muscle; *SSP*, supraspinatus muscle.

ISP, SSC, and SSP are rotator cuff muscles.

A post-hoc power analysis was performed. With a total sample size of 38, the achieved statistical power was 95.1%.

## Discussion

This study was conducted to clarify the association between RC muscle CSA and periscapular blood flow velocity by investigating the association between the PSV of the DSA and CSAs of the SSP, ISP, and SSC. The results showed that only the ISP measured at the GF level was significantly and negatively associated with PSV, indicating a relationship between the ISP muscle size and periscapular hemodynamics.

The previous study investigated the PSV of the DSA in patients with RCTs and showed that the PSV was 22.6 ± 7.4 cm/s on the affected side and 18.9 ± 6.9 cm/s on the unaffected side.^18^ This suggests an increase in blood flow on the affected side, which is also observed in the present study. Furthermore, when compared with previously reported RC muscle CSA in healthy individuals at the SS level (SSP: 841 ± 191 mm^2^, ISP: 1,568 ± 338 mm^2^, SSC: 2,343 ± 587 mm^2^),^4^ all muscles in the present study showed atrophy in the tear group. Similarly, at the GF level, CSA values in the current study were smaller than those reported previously (SSP: 4.0-4.2 cm^2^, ISP: 8.4-10.7 cm^2^, SSC: 7.9-9.4 cm^2^).^15^ Based on these results, the changes in PSV of the DSA and CSA observed in the patients of this study could be caused by RCTs.

Notably, the periscapular blood flow velocity was associated with ISP muscle size, despite the DSA not directly supplying the ISP. This finding suggests that atrophy of the ISP, which is considered important in determining the success of conservative treatment, may influence regional hemodynamics. Because this study employed an observational, cross-sectional design, the directionality of this association cannot be established. A plausible explanation is that ISP atrophy compromises glenohumeral stability, prompting compensatory hyperactivity in scapular muscles supplied by the DSA—such as the levator scapulae and rhomboids—which in turn elevates local blood flow. Although the exact mechanisms underlying this association remain unclear, a reduction in ISP muscle size may result in reduced glenohumeral stability, triggering compensatory overactivity of the surrounding periscapular muscles. Consequently, the high muscular demand may increase the PSV of the DSA, which supplies these muscles. Supporting this, a study that examined scapular upward rotation and humeral head translations before and after suprascapular nerve block reported a significant increase in both after the nerve block.^26^ Furthermore, another study revealed that ISP contraction suppresses anterior translation of the humeral head.^24^ These findings support, a previous study that used fluorodeoxyglucose positron-emission tomography/CT to evaluate periscapular muscle metabolism during shoulder AB in patients with massive RCTs showed that larger active AB ranges were accompanied by increased fluorodeoxyglucose uptake in the levator scapulae, rhomboids, pectoralis major, and teres major, indicating compensatory activation of these muscles.^5^ These observations suggest that periscapular musculature may help compensate for the glenohumeral instability induced by RC deficiency.

Such compensatory muscular activity increases metabolic demand, leading to high oxygen and nutrient delivery and removal of metabolic byproducts, resulting in increased local blood flow.^17^^,^^22^ In patients with RCTs, this may lead to high PSV in the DSA, which supplies muscles such as the levator scapulae and rhomboid muscles. Studies have demonstrated that the PSV of the transverse cervical artery, which supplies the trapezius muscle, is positively correlated with the stiffness of the trapezius muscle.^1^^,^^28^ Therefore, increased muscle stiffness or spasms in DSA-supplied muscles may cause local ischemia and sustained metabolic demand, resulting in increased PSV. Collectively, these findings indicate that ISP atrophy may indirectly influence the PSV of the DSA through the compensatory activity of the periscapular muscles. Nonetheless, because periscapular muscle activity was not directly assessed in the present study, these interpretations remain provisional. Longitudinal investigations that incorporate electromyographic assessment of muscle activity are required to clarify causal relationships.

In contrast, no significant associations were found between the PSV of the DSA and any RC muscles measured at the SS level. A study that assessed glenohumeral instability patterns and RC muscle size at the GF level reported severe SSC hypertrophy in patients with anterior instability but severe ISP hypertrophy in those with posterior instability.^15^ These findings suggest that the observed increase in PSV of the DSA may be more attributable to factors related to glenohumeral stability at the GF level, rather than to generalized RC muscle atrophy at the SS level.

Additionally, vascular structural changes associated with aging or obesity could contribute to muscle atrophy through impaired perfusion. However, in the present study, the CSAs of the SSP and SSC were not significantly associated with the PSV, and multiple regression analysis adjusted for age and BMI also identified only ISP as a significant predictor. If muscle atrophy were solely due to vascular structural changes related to aging or obesity, associations with SSP and SSC would also be expected, particularly in the GF or SS levels. Because only the ISP at the GF level was associated with PSV in this study, the observed association is less likely to be attributed to age- or obesity-related vascular changes.

Compared with nonspecific exercise programs, a scapula-focused exercise program for RC-related shoulder pain was reported to provide greater mid-term improvements in pain and function.^20^ This study suggests a significant association between the CSA of the ISP and the PSV of the DSA. Our findings indicate that effective rehabilitation may require not only strengthening the atrophied RC muscles but also reducing compensatory hyperactivity in muscles such as the rhomboids and levator scapulae, which may underlie the observed increases in PSV, through interventions such as manual therapy or relaxation training. Future studies are warranted to examine whether physical therapy strategies targeting vascular changes might be effective interventions for managing pain and functional deficits in RCTs.

This study has several limitations. First, although the association between CSA and PSV was examined, a previous study reported a positive correlation between muscle mass and vascular status.^14^ Age-related oxidative stress, inflammation, and hormonal dysregulation can induce endothelial dysfunction, potentially restricting blood flow and limiting nutrient delivery to skeletal muscle.^16^ Therefore, reduced perfusion may contribute to muscle atrophy. Given that the PSV reflects transient circulatory changes, it has limited capacity to assess structural vascular characteristics such as stiffness. Thus, to comprehensively evaluate the association between vascular function and muscle atrophy, future studies may benefit from incorporating additional vascular indices such as pulse-wave velocity. Second, although a significant association was found between PSV and CSA of the ISP, potential confounders such as muscle stiffness and compensatory activation of periscapular muscles were not fully controlled. Therefore, we were unable to demonstrate a relationship between increased blood flow and muscle activity. Future investigations incorporating ultrasound elastography or electromyographic analysis may provide additional insights. Third, this study discussed the possibility that ISP muscle atrophy contributes to glenohumeral instability and compensatory scapular activity; however, the degree of actual instability was not directly evaluated. Therefore, future studies should examine the causal association between humeral head instability and periscapular blood flow using diagnostic imaging.

Fourthly, the PSV could be affected by several factors, like blood pressure at the point, diabetes, muscle exertion just before. Therefore, future studies should clarify the factors associated with blood flow velocity. Fifthly, this study did not include a control group. Consequently, it is unclear whether the observed hemodynamic and muscle mass changes represent true pathology or merely the physiological variation expected in an age-matched population. Future research should incorporate an appropriately matched control cohort, adjusted for factors such as age and sex, to allow for more rigorous comparative analyses. Lastly, this study employed a retrospective cross-sectional design, which precludes conclusions about causality between hemodynamic changes and muscle atrophy. Thus, longitudinal or interventional studies are necessary to determine whether vascular dysfunction directly contributes to muscle loss. The relatively small sample size (n = 38) also limits the generalizability of the findings and may have influenced the statistical power of the multivariate analyses. Multicenter studies with larger cohorts are warranted to confirm these findings.

## Conclusion

This study investigated the association between RC muscle CSA and the PSV of the DSA, and the results indicate that an increase in the PSV may be associated with a decrease in the CSA of the ISP at the GF level. These results highlight the potential importance of incorporating vascular dynamics into clinical assessment and therapeutic strategies for patients with RCTs.

## Acknowledgments

The authors wish to thank their colleagues for their valuable insights and discussions that have contributed to this research.

## Disclaimers

Funding: No funding was disclosed by the authors.

Conflicts of interest: The authors, their immediate families, and any research foundation with which they are affiliated have not received any financial payments or other benefits from any commercial entity related to the subject of this article.
